# *rre37* Overexpression Alters Gene Expression Related to the Tricarboxylic Acid Cycle and Pyruvate Metabolism in *Synechocystis* sp. PCC 6803

**DOI:** 10.1155/2014/921976

**Published:** 2014-12-28

**Authors:** Hiroko Iijima, Atsuko Watanabe, Junko Takanobu, Masami Yokota Hirai, Takashi Osanai

**Affiliations:** RIKEN Center for Sustainable Resource Science, 1-7-22 Suehiro-cho, Tsurumi-ku, Yokohama, Kanagawa 230-0045, Japan

## Abstract

The tricarboxylic acid (TCA) cycle and pyruvate metabolism of cyanobacteria are unique and important from the perspectives of biology and biotechnology research. Rre37, a response regulator induced by nitrogen depletion, activates gene expression related to sugar catabolism. Our previous microarray analysis has suggested that Rre37 controls the transcription of genes involved in sugar catabolism, pyruvate metabolism, and the TCA cycle. In this study, quantitative real-time PCR was used to measure the transcript levels of 12 TCA cycle genes and 13 pyruvate metabolism genes. The transcripts of 6 genes (*acnB*, *icd*, *ppc*, *pyk1*, *me*, and *pta*) increased after 4 h of nitrogen depletion in the wild-type GT strain but the induction was abolished by *rre37* overexpression. The repression of gene expression of *fumC, ddh*, and *ackA* caused by nitrogen depletion was abolished by *rre37* overexpression. The expression of *me* was differently affected by *rre37* overexpression, compared to the other 24 genes. These results indicate that Rre37 differently controls the genes of the TCA cycle and pyruvate metabolism, implying the key reaction of the primary in this unicellular cyanobacterium.

## 1. Introduction

The tricarboxylic acid (TCA) cycle and pyruvate metabolism are conserved in almost all organisms and are indispensable for cell survival and proliferation. Cyanobacteria were thought to have an incomplete TCA cycle because they lack the 2-oxoglutarate (2-OG) dehydrogenase enzyme [1], but Zhang and Bryant detected a closed and complete TCA cycle in the unicellular cyanobacterium* Synechococcus* sp. PCC 7002 [2]. In this cyanobacterium, 2-OG is converted to succinic semialdehyde, a step catalyzed by a 2-OG decarboxylase, and succinic semialdehyde is converted to succinate by a succinic semialdehyde dehydrogenase [2]. Genes encoding these two enzymes are conserved among cyanobacteria, except in marine species, indicating that the closed TCA cycle is widely maintained in cyanobacteria.

2-OG is a known signaling metabolite in cyanobacteria; its level increases by nitrogen depletion [3]. 2-OG directly interacts with a transcription factor NtcA, promoting its interaction with promoter DNAs and transcription activation of nitrogen-related genes by NtcA [4, 5]. 2-OG also binds to PII protein, which is a carbon/nitrogen balance sensor, and transduces nitrogen starvation signals [6, 7]. Metabolomic analyses have revealed that, in addition to 2-OG, other organic acids in the TCA cycle, including succinate, malate, and fumarate, are increased by nitrogen depletion in the unicellular cyanobacterium* Synechocystis* sp. PCC 6803 (hereafter referred to as* Synechocystis* 6803) and halophilic cyanobacterium* Arthrospira platensis *[8–10]. Amino acids derived from pyruvate metabolism and the TCA cycle metabolites, such as alanine, phenylalanine, tyrosine, serine, glycine, valine, and leucine, increase by nitrogen depletion, whereas aspartate, arginine, glutamate, and glutamine decrease [10]. Like glycogen and polyhydroxybutyrate, the pool of organic acids in the TCA cycle may function as carbon storage during nitrogen starvation [10]. The redistribution of carbon sources to various metabolites other than glycogen has been shown in the freshwater blooming cyanobacterium* Microcystis aeruginosa* PCC 7806 [11].

The expression of the gene encoding a response regulator Rre37 (sll1330) is induced by nitrogen depletion [12]. In the nitrogen-fixing cyanobacterium,* Anabaena* sp. PCC 7120, the expression of an ortholog of Rre37, named NrrA, also increases during nitrogen starvation and NtcA binds to the promoter region of* nrrA* [13, 14]. NrrA regulates glycogen catabolism by controlling the transcription of genes encoding the glycogen catabolism-related enzymes, glycolytic enzymes, and a group 2 sigma factor SigE [15].* Synechocystis* 6803 Rre37 affects the expression of genes encoding enzymes involved in glycolysis and glycogen catabolism [16, 17].* Synechocystis* 6803 NtcA binds to the promoter region of* rre37* in a 2-OG dependent manner* in vitro* [17]. Rre37 binds to the promoter regions of genes encoding enzymes involved in glycogen catabolism, glycolysis, and amino acid metabolism [18, 19]. Metabolomic analysis has shown that* rre37* knockout alters the levels of glycogen, sugar phosphates, and organic acids in the TCA cycle [20]. Recent genetic engineering studies using the* rre37*-overexpressing strain ROX370 showed that* rre37* overexpression decreased the levels of glycogen, sugar phosphates, and organic acids in the TCA cycle but increased glycogen catabolism-related enzymes, glycolytic enzymes, and polyhydroxybutyrates [19]. Combining transcriptomic and metabolomic analyses using ROX370 suggests that a possible new cycle, a hybrid of the TCA and ornithine cycle, may be induced during nitrogen starvation [19]. This hybrid cycle enables* Synechocystis *cells to assimilate two molecules of ammonium ions, leading to efficient nitrogen uptake during nitrogen starvation [19]. Thus, the integrity of the TCA cycle and pyruvate metabolism is important for survival during nitrogen starvation, where Rre37 may play a pivotal role in their regulation.

In this study, we examined the expression levels of genes related to the TCA cycle and pyruvate metabolism by quantitative real-time PCR. We found altered levels of transcript levels in response to the nitrogen status, mediated by* rre37* overexpression.

## 2. Materials and Methods

### 2.1. Bacterial Strains and Culture Conditions

The glucose-tolerant (GT) strain of* Synechocystis *sp. PCC 6803 [21] and ROX370 [19] were grown in modified BG-11 medium, which consisted of BG-11_0_ liquid medium [22] containing 5 mM NH_4_Cl (buffered with 20 mM Hepes-KOH, pH 7.8). The GT-I strain was used in this study [23]. Liquid cultures were bubbled with 1% (v/v) CO_2_ in air and incubated at 30°C under continuous white light (approx. 50–70 *μ*mol photons m^−2^ sec^−1^). Growth and cell densities were measured at A_730_ with a Hitachi U-3310 spectrophotometer (Hitachi High-Tech., Tokyo, Japan).

### 2.2. RNA Isolation and Quantitative Real-Time PCR

Cells were diluted to A_730_ = 0.2 in 70 mL of modified BG-11 medium and cultivated overnight. Nitrogen sources were depleted from the medium by filtering the cells and suspending them in BG-11_0_ medium. RNA isolation, cDNA synthesis, and quantitative real-time PCR were performed as described previously [10]. The primers used for quantitative real-time PCR are listed in Table 1.

## 3. Results

### 3.1. Transcript Levels of Genes Related to the TCA Cycle in ROX370

**Figure 1:**
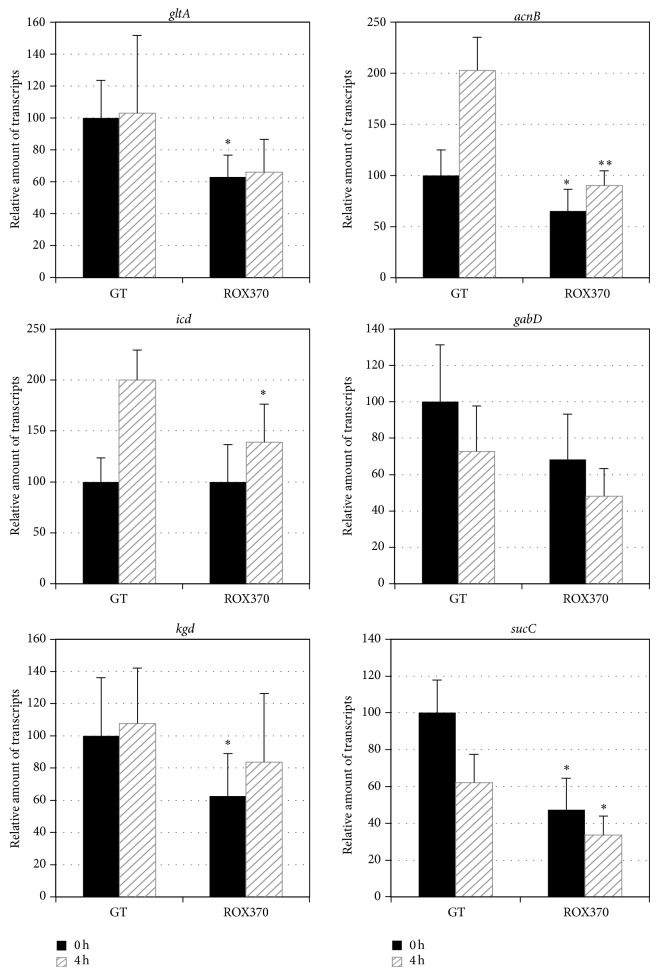
Quantitative real-time PCR analysis of transcription in GT and ROX370. Relative transcript levels of 6 genes involved in the TCA cycle pathway (*gltA*,* acnB*,* icd*,* gabD*,* kdg*, and* sucC*) are described. Data represent the mean ± SD from four independent experiments. Transcript levels were calibrated relative to that of corresponding levels in GT under nitrogen-replete conditions (set at 100%). Asterisks indicate statistically significant differences between GT and ROX370 (Student's *t*-test; ^*^ *P* < 0.05, ^**^ *P* < 0.005).

**Figure 2:**
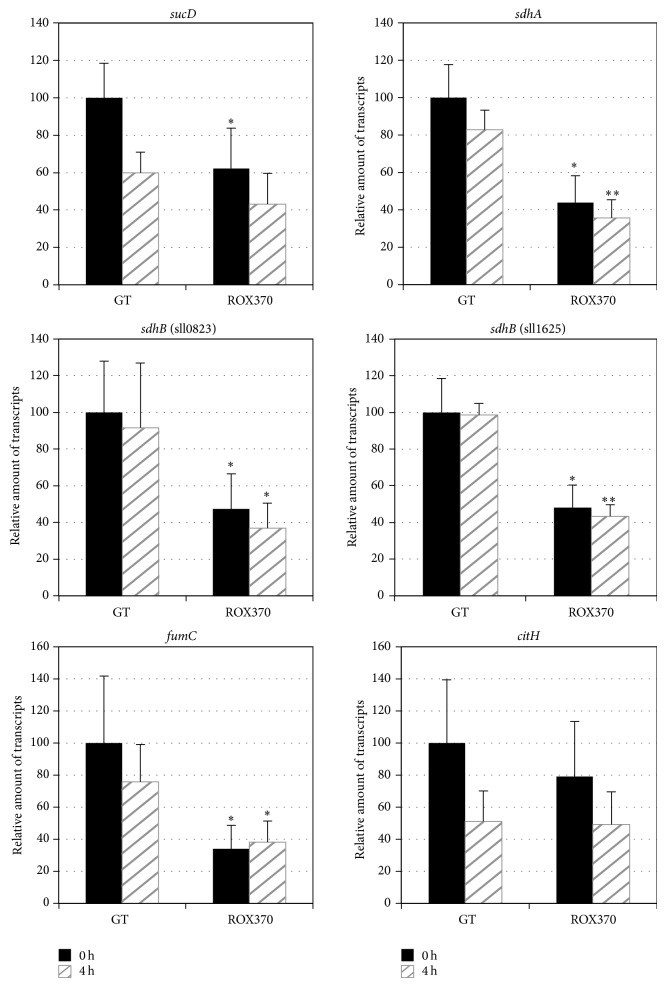
Quantitative real-time PCR analysis of transcription in GT and ROX370. Relative transcript levels of 6 genes involved in the TCA cycle pathway (*sucD*,* sdhA*,* sdhB* (sll0823),* sdhB* (sll1625),* fumC*, and* citH*) are described. Data represent the mean ± SD from four independent experiments. Transcript levels were calibrated relative to that of corresponding levels in GT under nitrogen-replete conditions (set at 100%). Asterisks indicate statistically significant differences between GT and ROX370 (Student's *t*-test; ^*^ *P* < 0.05, ^**^ *P* < 0.005).

The transcripts of 12 genes related to the TCA cycle,* gltA* (sll0401, encoding a citrate synthase),* acnB* (slr0665, encoding an aconitate hydratase),* icd* (slr1289, encoding an isocitrate dehydrogenase),* gabD* (slr0370, encoding a succinic semialdehyde dehydrogenase),* kgd* (sll1981, encoding a 2-OG decarboxylase),* sucC* (sll1023, encoding a succinyl-CoA synthetase beta chain),* sucD* (sll1557, encoding a succinyl-CoA synthetase alpha chain),* sdhA* (slr1233, encoding a succinate dehydrogenase flavoprotein subunit),* sdhB* (sll0823) (encoding a succinate dehydrogenase iron-sulfur subunit),* sdhB* (sll1625) (encoding a succinate dehydrogenase iron-sulfur subunit),* fumC* (slr0018, encoding a fumarase), and* citH* (sll0891, encoding a malate dehydrogenase), were measured using cells grown under nitrogen-replete conditions or nitrogen-depleted conditions for 4 h. The expression of* acnB* and* icd* increased in the GT strain after 4 h of nitrogen depletion (Figure 1).* rre37* overexpression abolished the induction of* acnB *and* icd* expression under nitrogen-depleted conditions (Figure 1). The expression of* kgd* in ROX370 was lower than that of the GT strain under nitrogen-replete conditions (Figure 1). The transcripts of* gabD*,* sucC*,* sucD*,* fumC*, and* citH* in the GT strain decreased by nitrogen-depleted conditions (Figures 1 and 2). A decrease in the levels of transcripts, except* fumC*, was also observed in ROX370 strain after nitrogen starvation (Figure 2). The expression of genes encoding the succinate dehydrogenase subunits,* sdhA*,* sdhB* (sll0823), and* sdhB* (sll1625), was lower in ROX370 than in the GT strain under both nitrogen-replete and nitrogen-depleted conditions (Figure 2).

### 3.2. Transcript Levels of Genes Related to the Pyruvate Metabolism in ROX370

**Figure 3:**
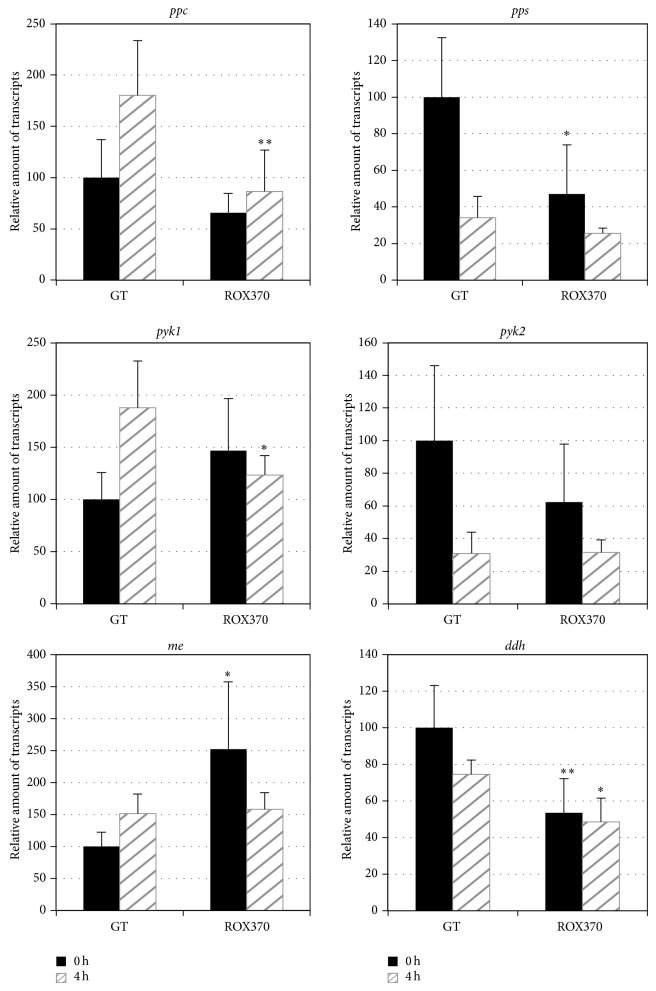
Quantitative real-time PCR analysis of transcription in GT and ROX370. Relative transcript levels of 6 genes involved in pyruvate metabolism (*ppc*,* pps*,* pyk1*,* pyk2*,* me*, and* ddh*) are shown. Data represent the mean ± SD from four independent experiments. Transcript levels were calibrated relative to that of corresponding levels in GT under nitrogen-replete conditions (set at 100%). Asterisks indicate statistically significant differences between GT and ROX370 (Student's *t*-test; ^*^ *P* < 0.05, ^**^ *P* < 0.005).

**Figure 4:**
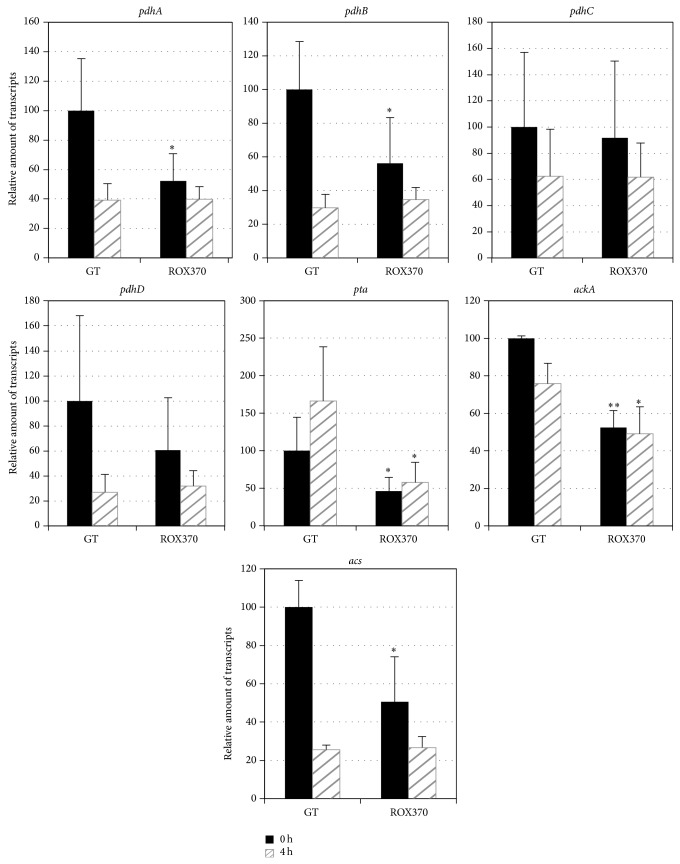
Quantitative real-time PCR analysis of transcription in GT and ROX370. Relative transcript levels of 7 genes involved in pyruvate metabolism (*pdhA*,* pdhB*,* pdhC*,* pdhC*,* pta*,* ackA*, and* acs*) are shown. Data represent the mean ± SD from four independent experiments. Transcript levels were calibrated relative to that of corresponding levels in GT under nitrogen-replete conditions (set at 100%). Asterisks indicate statistically significant differences between GT and ROX370 (Student's *t*-test; ^*^ *P* < 0.05, ^**^ *P* < 0.005).

The transcripts of 13 genes related to the pyruvate metabolism,* ppc* (sll0920, encoding a phosphoenolpyruvate carboxylase),* pps* (slr0301, encoding a phosphoenolpyruvate synthase),* pyk1* (sll0587, encoding a pyruvate kinase),* pyk2* (sll1275, encoding a pyruvate kinase),* me* (slr0721, encoding a malic enzyme),* ddh* (slr1556, encoding a D-lactate dehydrogenase),* pdhA* (slr1934, encoding a subunit of pyruvate dehydrogenase),* pdhB* (sll1721, encoding a subunit of pyruvate dehydrogenase),* pdhC* (sll1841, encoding a subunit of pyruvate dehydrogenase),* pdhD* (slr1096, encoding a subunit of pyruvate dehydrogenase),* pta* (slr2131, encoding a phosphoacetyltransferase),* ackA* (sll1299, encoding an acetate kinase), and* acs *(sll0542, encoding an acetyl-CoA synthetase), were measured using cells grown under nitrogen-replete conditions or nitrogen-depleted conditions for 4 h. The expressions of* ppc*,* pyk1*,* me*, and* pta* increased after 4 h of nitrogen depletion in the GT strain, but the increases were abolished by* rre37* overexpression (Figures 3 and 4). In particular,* rre37 *overexpression affected the expression of* pyk1* and* me*, whose transcripts increased in the GT strain but decreased in the ROX370 strain after nitrogen depletion (Figure 3). The transcript levels of* pps*,* pyk2*,* ddh*,* pdhABCD*,* ackA*, and* acs* decreased in the GT strain by nitrogen depletion; the levels of these transcripts, except for* ddh* and* ackA*, also decreased in the ROX370 strain (Figures 3 and 4).

## 4. Discussion

Quantitative real-time PCR analysis demonstrated that the levels of transcripts of 6 genes related to the TCA cycle and pyruvate metabolism increased under conditions of nitrogen depletion in the wild-type strain, but these increases were abolished by* rre37* overexpression (Figures 1–4). The results showed that the transcript levels of the genes whose expression is induced by nitrogen depletion particularly were altered by* rre37 *overexpression (Figures 1, 3, and 4). These results indicate that the proper amount of Rre37 proteins is important in the transcriptional activation of these genes during nitrogen starvation, consistent with a previous study [17]. Among the 25 genes tested in this study, the transcription of* icd* was activated by NtcA [24], and the other 24 genes did not seem to be regulated by NtcA [25]. A previous study suggested that transcription of* me* is positively regulated by Rre37, although further biochemical analysis is required to validate this [19]. In addition to* in vitro* analysis, chromatin immunoprecipitation (ChIP) showed that, in* Anabaena *sp. PCC 7120, 55 genes involved in primary metabolism, including* pps*, are potentially regulated by NtcA [26]. Using genetic analyses, we demonstrated that Rre37 is involved in the transcriptional regulation of the genes related to the TCA cycle and pyruvate metabolism. Future studies with ChIP analyses may uncover whether Rre37 regulates genes in the TCA cycle and pyruvate metabolism directly or indirectly.

The expression of* rre37* is affected by environmental conditions and genetic modifications. SigE is a global regulator of sugar catabolism activation [27, 28], and* sigE* overexpression increases polyhydroxybutyrate and hydrogen production [29, 30]. Rre37 protein levels are upregulated by* sigE* knockout [17]. Another study has shown that knockout of a gene encoding the transcription factor SyAbrB2 decreases the transcript levels of* sigE*,* gnd* (encoding 6-phosphogluconate dehydrogenase),* pyk1*,* pyk2*, and* icd* [31], implying that Rre37 levels may be affected by* SyabrB2* knockout. Thus, several transcriptional regulators, including NtcA, SigE, and SyAbrB2, are involved in the regulation of* rre37* transcription either directly or indirectly. Imamura et al. demonstrated that sigma factors are differently associated with the promoters of nitrogen-related genes in response to growth phase and external nutrient conditions [32]. The role of PipX, a small protein binding to NtcA in a 2-OG dependent manner [33], should be considered in* rre37* transcription during nitrogen starvation. Thus, multiple factors control Rre37 levels, leading to the regulation of primary metabolism in* Synechocystis *6803. Elucidation of the regulatory mechanism of* rre37* transcription is complicated but is necessary for understanding of the primary metabolism of* Synechocystis* 6803. This is because transcript analyses have revealed that multiple transcriptional regulators, including Rre37, are involved in regulating the expression of metabolic enzymes, as also evidenced in this study.

**Figure 5:**
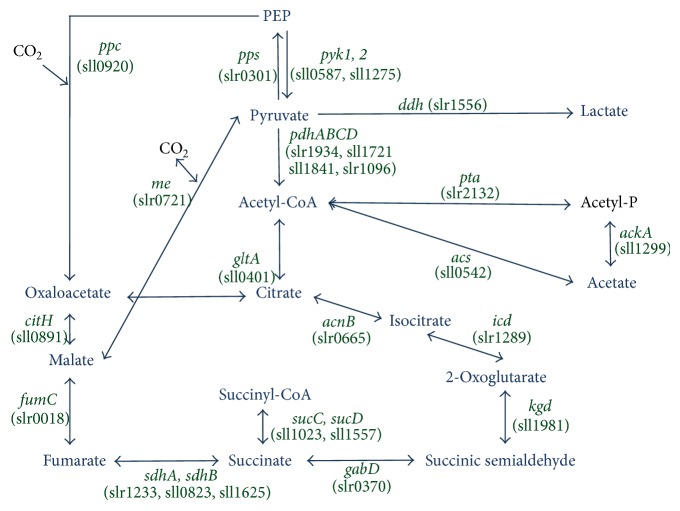
The metabolic map around the TCA and pyruvate metabolism in* Synechocystis* 6803.

We analyzed ROX370, overexpressing* rre37* strain, and found that the expression of genes related to primary metabolism is widely affected (Figures 1–4) [19]. We could not distinguish between the direct and indirect effects of* rre37* overexpression in the altered transcription seen in the ROX370 strain. Therefore, we could not elucidate the molecular mechanisms underlying transcriptional regulation by Rre37. However, these results demonstrate that genetic engineering of a transcription factor rewires* Synechocystis* 6803 metabolism, which is beneficial for biotechnological applications for producing high-value products. In addition, the expression pattern of* me* was different from the other 24 genes tested (Figures 1–4), and these results may indicate the key enzyme of the primary metabolism in this cyanobacterium. Accumulation of organic acids in the later half of the TCA cycle during nitrogen starvation has been demonstrated [10], and the transcript results suggest that the malic enzyme may play important roles in their accumulation (Figure 5). The metabolomic technique is indispensable for the analysis of these mutants and provides significant information regarding primary metabolism in* Synechocystis* 6803 as previously described [10, 28]. This is significant, considering that both carbon and nitrogen signals mediated by transcription factors are important in the regulation of primary metabolism and carbon uptake in other cyanobacteria [34].

## 5. Conclusion

The TCA cycle and pyruvate metabolism are important for interaction between carbon and nitrogen metabolism. Detailed analysis of their regulatory mechanisms is valuable in understanding the biology of metabolism of cyanobacteria, in biotechnological applications for producing valuable products [35], and in environmental microbiology for avoiding water pollution [36]. The transcript levels analysis in this study demonstrated the differential control of the transcript levels of the TCA and pyruvate metabolism, possibly indicating key enzymes in primary metabolism of this unicellular cyanobacterium.

## Figures and Tables

**Table 1:** Primer list for quantitative real-time PCR.

Gene name	
*rnpB *	Forward primer 5′-AAAGGGTAAGGGTGCAAAGG-3′
	Reverse primer 5′-AATTCCTCAAGCGGTTCCAC-3′
*gltA *	Forward primer 5′-ATCGAGGGTGAGCCATGTG-3′
	Reverse primer 5′-GCGAATGCCCCGGTACT-3′
*acnB *	Forward primer 5′-TCACCCTCGCCCAAAAAA-3′
	Reverse primer 5′-GTGCCGGGACGAATACCTT-3′
*icd *	Forward primer 5′-CCCCGGCTCTGTGATCCT-3′
	Reverse primer 5′-TGCCAGCCCATAAATTCCA-3′
*gabD *	Forward primer 5′-TGCGCAAGTAGAACAAACCATT-3′
	Reverse primer 5′-TGGCCGCCACAACGA-3′
*kgd *	Forward primer 5′-CCATTTCCAAGGCCAAAAAC-3′
	Reverse primer 5′-GCTTCGGCTCGGATGGT-3′
*sdhA *	Forward primer 5′-GTCTGGCCCCTGATACCAAA-3′
	Reverse primer 5′-GAACGGATGGGATGGGTTT-3′
*sdhB*(sll0823)	Forward primer 5′-TCAGATCAAATGGCAACAGGAT-3′
	Reverse primer 5′-TGGCATTACGGCAATTCTTG-3′
*sdhB*(sll1625)	Forward primer 5′-TGCAGTATGCGGGTTAATGG-3′
	Reverse primer 5′-TTCACTGCCCACATTTTCCTT-3′
*sucC *	Forward primer 5′-CCCTCAAACGGTTGCAAATT-3′
	Reverse primer 5′-GCCCGCACCTGGGATT-3′
*sucD *	Forward primer 5′-GGGCGCAAAAATCAAACG-3′
	Reverse primer 5′-AGTTGGTTGGCCACAATGGT-3′
*fumC *	Forward primer 5′-GAATGTTTTGCAGGCATCACTAAA-3′
	Reverse primer 5′-GGGCACTGCGTCCATCA-3′
*citH *	Forward primer 5′-CTGAAATTGCCGCCTTACTACA-3′
	Reverse primer 5′-AAGAGGCCGGCGCATAA-3′
*ppc *	Forward primer 5′-CCACCACCACAGCCCTACTAA-3′
	Reverse primer 5′-GTCGGAATAGCCCACCATAATTT-3′
*pps *	Forward primer 5′-TCACTGACCGGGCTATTTCCT-3′
	Reverse primer 5′-CCACCGCAAAATGGTCAAA-3′
*pyk1 *	Forward primer 5′-CGTGGCCAACGCTATTTTG-3′
	Reverse primer 5′-CGATTCCCCCGATAACATCA-3′
*pyk2 *	Forward primer 5′-ATGCCGGCTCTGTGCAA-3′
	Reverse primer 5′-GGGCGACTGGTGAGGGTAT-3′
*me *	Forward primer 5′-CGGAGCCACCGATATTTGG-3′
	Reverse primer 5′-TGCGATGTTTGCCCACAA-3′
*ddh *	Forward primer 5′-AGCAAACCACCCCCATCA-3′
	Reverse primer 5′-CAAGGTTGAGTTGGGCATCA-3′
*pdhA *	Forward primer 5′-CACGAGCGGGCAACGT-3′
	Reverse primer 5′-TGTTGAACACACTGGCTTTTTTG-3′
*pdhB *	Forward primer 5′-CCGCATGCGTCACCATT-3′
	Reverse primer 5′-GGTCGTAGCCTTCTTTTTCCAA-3′
*pdhC *	Forward primer 5′-GGGCAACCCTTGGCCTAGT-3′
	Reverse primer 5′-GCTTGGGCTTCGGCAAT-3′
*pdhD *	Forward primer 5′-AAAATCCAATCTGACCTGACCAA-3′
	Reverse primer 5′-CCCCGGATGGTATCGACTT-3′
*pta *	Forward primer 5′-GACGCCCCTCCCCTGTT-3′
	Reverse primer 5′-AAACGGGCGGAAGTTTCAT-3′
*ackA *	Forward primer 5′-CCTGGTGGGCCATCGA-3′
	Reverse primer 5′-AAAGTGGCTTCGGCATGATC-3′
*acs *	Forward primer 5′-TGGCGGCGGTAAATGC-3′
	Reverse primer 5′-CGCCGGTTTCCGTTTG-3′
